# Porous Lithium Disilicate Glass–Ceramics Prepared by Cold Sintering Process Associated with Post-Annealing Technique

**DOI:** 10.3390/ma17020381

**Published:** 2024-01-12

**Authors:** Xigeng Lyu, Yeongjun Seo, Do Hyung Han, Sunghun Cho, Yoshifumi Kondo, Tomoyo Goto, Tohru Sekino

**Affiliations:** 1 SANKEN (The Institute of Scientific and Industrial Research), Osaka University, 8-1 Mihogaoka, Ibaraki 567-0047, Osaka, Japan; lxgeng23423@sanken.osaka-u.ac.jp (X.L.); yjseo@sanken.osaka-u.ac.jp (Y.S.); dh.han23@sanken.osaka-u.ac.jp (D.H.H.); shcho@sanken.osaka-u.ac.jp (S.C.); y.kondo@sanken.osaka-u.ac.jp (Y.K.); goto@sanken.osaka-u.ac.jp (T.G.); 2 Institute for Advanced Co-Creation Studies, Osaka University, 1-1 Yamadaoka, Suita 565-0871, Osaka, Japan

**Keywords:** cold sintering process, lithium disilicate, porous glass–ceramics, mechanical properties

## Abstract

Using melt-derived LD glass powders and 5–20 M NaOH solutions, porous lithium disilicate (Li_2_Si_2_O_5_, LD) glass–ceramics were prepared by the cold sintering process (CSP) associated with the post-annealing technique. In this novel technique, H_2_O vapor originating from condensation reactions between residual Si–OH groups in cold-sintered LD glasses played the role of a foaming agent. With the increasing concentration of NaOH solutions, many more residual Si–OH groups appeared, and then rising trends in number as well as size were found for spherical pores formed in the resultant porous LD glass–ceramics. Correspondingly, the total porosities and average pore sizes varied from 25.6 ± 1.3% to 48.6 ± 1.9% and from 1.89 ± 0.68 μm to 13.40 ± 10.27 μm, respectively. Meanwhile, both the volume fractions and average aspect ratios of precipitated LD crystals within their pore walls presented progressively increasing tendencies, ranging from 55.75% to 76.85% and from 4.18 to 6.53, respectively. Young’s modulus and the hardness of pore walls for resultant porous LD glass–ceramics presented remarkable enhancement from 56.9 ± 2.5 GPa to 79.1 ± 2.1 GPa and from 4.6 ± 0.9 GPa to 8.1 ± 0.8 GPa, whereas their biaxial flexural strengths dropped from 152.0 ± 6.8 MPa to 77.4 ± 5.4 MPa. Using H_2_O vapor as a foaming agent, this work reveals that CSP associated with the post-annealing technique is a feasible and eco-friendly methodology by which to prepare porous glass–ceramics.

## 1. Introduction

Lithium disilicate (Li_2_Si_2_O_5_, written as LD) glass–ceramics, the microstructure of which features interlocked rod-like LD crystals embedded in glass matrix [1,2,3], have gained much attention in prosthetic dentistry since 1998 [4]. In recent years, a novel restorative material, polymer-infiltrated-ceramic-network material (PICN), has gained researchers’ attention [5,6,7]. It features excellent mechanical properties [5,6] through interpenetrating polymers into porous ceramics. Until now, polymer-infiltrated porous feldspar ceramics [7], polymer-infiltrated porous zirconia ceramics [6], and other porous ceramics systems [5,8] have been successfully prepared. However, there has not been a report regarding polymer-infiltrated porous LD glass–ceramics. It is urgent to prepare porous LD glass–ceramics, and we expect to obtain polymer-infiltrated porous LD glass–ceramics with satisfying mechanical properties, which would broaden the practical applications of LD glass–ceramics. Furthermore, studies have reported that LD glass–ceramics has potential applications in pyroelectrics [9] due to the unique sandwich crystal structure of LD crystals, in which Li^+^ ions exhibit mobility in the dimensional direction among corrugated [SiO_4_] layers [10]. In addition to crystalline phases of LD glass–ceramics, Li^+^ ions also possess better diffusion abilities within the amorphous LD phase [11]. Thus, LD glass–ceramics, possessing excellent Li^+^ ion migration abilities, have great potential applications in lithium ions batteries such as solid-state electrolytes [11] and inorganic separators [12,13]. For instance, D. Li et al. applied a high-temperature solid-state reaction method to prepare a porous lithium silicate ceramic separator [12,13], which was constructed by three-dimensional pore structures, and an inorganic matrix consisting of functional LD crystals. The resultant lithium ion batteries exhibited excellent performances compared to commercial polyolefin separators [12,13]. It is a pity that the ununiformly distributed pore structures inevitably weakened the performance of the resultant lithium ion batteries [14,15]. Hence, it is necessary to develop a more advanced technique to prepare porous LD glass–ceramics possessing more uniform pore structures. H. Zhang et al. also found that LD crystals had great advantages for the effective adsorption of heavy metal ions [16,17] and methylene blue [10,18], indicating their potential use in wastewater treatment. However, developed LD-related materials only include nanomaterials [10,16,18], and LD nano-brush-coated cloths [17]. The lack of a self-supporting structure limits their durability in industrial applications. Thus, the preparation of porous LD glass–ceramics, working as self-supporting absorbents, is still necessary. In addition to the above discussion, it is urgent to develop porous LD glass–ceramics with uniform pore structures, which have exhibited great potential in the fields of dental restoration, lithium ion batteries, and wastewater treatment.

Recently, the cold sintering process (CSP), featuring energy-saving and low CO_2_ emission properties [19], was utilized to prepare porous ceramic materials. For instance, porous alumina ceramics with uniform structures were fabricated with NaCl as the pore-forming agent [20] using CSP and the post-annealing technique. Microporous TiO_2_ materials were prepared through CSP associated with the post-annealing technique [21], using thermoplastic polymer beads as sacrificial templates. Meanwhile, porous TiO_2_-reduced graphene oxide (TiO_2_/rGO) nanocomposites with evenly distributed pores were synthesized using polymethyl methacrylate microspheres as sacrificial templates [22] though CSP associated with the post-annealing technique. These research results suggest that easily-operated CSP associated with the post-annealing technique was helpful in preparing porous ceramics with uniform pore distribution due to the evenly distributed foaming agents in cold-sintered ceramic materials. This novel technique can be expected to obtain porous LD glass–ceramics with uniform pore structures through introducing proper foaming agents into cold-sintered LD glass materials.

Meanwhile, NaOH solutions have been widely utilized as transient solvents to aid in the densification process of cold-sintered glass materials [23,24,25,26], for which dissolution–precipitation processes were utilized according to the depolymerization and condensation reactions induced by NaOH solutions, as shown in Equations (1) and (2).

(1) $$\equiv \mathrm{Si}-O-\mathrm{Si}\equiv +\mathrm{NaOH}\to \equiv {\mathrm{Si}-O}^{-}{\mathrm{Na}}^{+}+\equiv \mathrm{Si}-\mathrm{OH}$$

(2) $$\equiv \mathrm{Si}-\mathrm{OH}+\equiv \mathrm{Si}-\mathrm{OH}\to \equiv \mathrm{Si}-O-\mathrm{Si}\equiv +H_{2}O↑$$

Moreover, Yanagisawa et al. [27] pointed out that H_2_O incorporated into glass structures could act as an effective foaming agent in the preparation of porous glass–ceramics. In their work, raw glass powder was subjected to hydrothermal treatment, and then incorporated H_2_O was released as vapor to form pores in prepared glass–ceramics with further heat treatment. Inspired by this reported work, we considered that H_2_O vapor originating from the residual Si–OH groups (shown in Equation (2)) of cold-sintered glasses may induce the formation of uniform pores in heat-treated glass–ceramics. In other words, not only can alkali solutions act as transient solvents to promote the densification process of cold-sintered glasses, but they also possibly supplied H_2_O vapor as a foaming agent to induce pore formation for the prepared glass–ceramics. Unlike introducing additional foaming agents such as SiC and CaCO_3_ [28,29], for which generated CO_2_ gas has been used to trigger the pore formation, the usage of H_2_O vapor as a foaming agent can be expected to be more eco-friendly.

In the current work, we attempted to apply CSP associated with post-annealing techniques to prepare porous LD glass–ceramics. For this purpose, melt-derived LD glass powders and 5–20 M NaOH solutions were selected as raw materials. To clarify the effects of the concentration of NaOH solution on both the foaming and crystallizing processes of post-annealed porous LD glass–ceramics, the silicate structures and thermal behaviors of cold-sintered LD glasses were investigated. The relative densities, total porosities, phase structures, and microstructures of cold-sintered LD glasses were compared to those of post-annealed porous LD glass–ceramics. In addition, the influences of the pores formed and the LD crystals precipitated in the pore walls on the mechanical properties of porous LD glass–ceramics were characterized.

## 2. Materials and Methods

### 2.1. Preparation of Lithium Disilicate (Li_2_Si_2_O_5_, LD) Glass Powders

LD glass material, the nominal composition of which was 68.6% SiO_2_, 28.6% Li_2_O, 2.0% K_2_O, and 0.8% La_2_O_3_ (in mol.%), was utilized in current work. According to the phase diagram of the Li_2_O–SiO_2_ system [30] and related research [2,31], the molar ratio of SiO_2_:Li_2_O for LD glass materials was determined to be 2.39:1 for the current research, which was beneficial for the precipitation of LD crystals after proper heat treatment. The K_2_O component was used to decrease the melting temperature [31] in order to prepare the LD glass materials. The La_2_O_3_ component was introduced to decrease the viscosity of the LD glass matrix [31], which was beneficial for the foaming process of the resultant porous LD glass–ceramics [32]. This multicomponent glass material was prepared by homogenizing a mixture of regent-grade SiO_2_, LiCO_3_, K_2_CO_3_, and La_2_O_3_ (purity > 99.9%, Sinopharm Chemical Reagent Co., Ltd., Shanghai, China). The obtained mixtures were melted in silica crucible at 1600 °C for 4 h in air, then immediately quenched in deionized water to prepare glass frits. Glass frits were ball-milled with high-purity zirconia balls for 10 h in an ethanol (EtOH, 99.5%, FUJIFILM Wako Pure Chemical Corp., Osaka, Japan) environment, and LD glass powders were obtained after sieving with a 75 μm sieve (IIDA Manufacturing Co., Ltd., Osaka, Japan) for the following experiments.

### 2.2. CSP Associated with Post-Annealing Process

**Figure 1:**
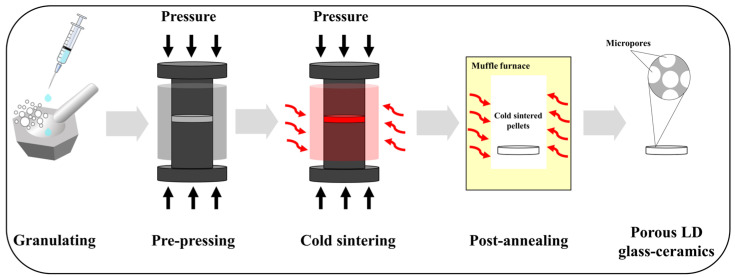
Schematic diagrams of the overall experimental procedure for preparing porous LD glass–ceramics.

LD glass powders (0.4 g) were mixed with 0.1 g of 5–20 M NaOH solutions prepared using NaOH flakes (FUJIFILM Wako Pure Chemical Corporation, Osaka, Japan). The mixture was placed into a mold with diameter of 15 mm and pressed under a uniaxial pressure of 350 MPa. Simultaneously, the mold was heated to 200 °C with a heating rate of 10 °C/min and held for 30 min. Then, the pressure was released gradually along with natural cooling. Pellets, taken out from the mold, were washed with absolute ethanol and kept in the oven at 60 °C for 24 h. The post-annealing process was undertaken in a muffle furnace (EPDS-7.2K, ISUZU SEISAKUSHO Co., Ltd., Niigata, Japan). Under a heating rate of 5 °C/min, cold-sintered pellets were post-annealed at 800 °C for 30 min. The prepared pellets were notated as shown in Table 1, and detailed schematic diagrams of the overall experimental procedure for preparing porous LD glass–ceramics are presented in Figure 1.

### 2.3. Characterization

Cold-sintered bulk samples were ground into fine powers for the purpose of characterizing their silicate structures and thermal behaviors [33]. The solid-state magic-angle spinning nuclear magnetic resonance technique (MAS NMR, AVANCE III 600 WB, Bruker BioSpin GmbH, Rheinstetten, Germany) was applied to detect silicate structures. ^29^Si MAS NMR spectra were measured at 119.25875 MHz (14.0989 T) using single-pulse excitation with a π/2 pulse time 4.5 μs in length, relaxation delays of 60 s, and scans of 320. Powdered samples were spun at the magic angle at a rate of 12 kHz within 4 mm zirconia rotors. ^1^H MAS NMR spectra were measured at 600.28 MHz (14.0989 T) using single-pulse excitation with a π/2 pulse time 3.0 μs in length, relaxation delays of 5 s, and scans of 64. Powdered samples were spun at the magic angle at a rate of 15 kHz within 4 mm zirconia rotors. All the chemical shifts were externally referenced to tetramethylsilane. The Q*^n^* distributions were obtained by curve fitting, and quantification of Q*^n^* structural units was obtained by assuming a Gaussian distribution contribution of each Q*^n^* specie to the total spectra of ^29^Si [34] using Delta 5.3.1 software. Thermal behaviors were analyzed by thermo gravimetric analysis (TGA) and differential thermal analysis (DTA) (TG-DTA, TG8120, RIGAKU Corp., Tokyo, Japan) in an air atmosphere from room temperature to 1200 °C at a heating rate of 20 °C/min. Phase structures of the bulk samples were detected by X-ray diffraction (XRD, D8 ADVANCCE, Bruker AXS Co., Ltd., Karlsruhe, Germany) using CuKα radiation. XRD was performed by measuring 2*θ* from 20° to 60° at a step size of 0.02°, with a step time of 0.2 s. Volume fractions of crystal phases were calculated by fitting full-spectrum XRD through Rietveld refinement using Jade 6.5 software [35]. Bulk densities were measured by Archimedes’ method using absolute ethanol as liquid medium, then converted to relative densities using the theoretical densities of LD glass or glass–ceramics [2]. Fractured surfaces were observed using a field-emission scanning electron microscope (FE-SEM, SU9000, Hitachi High-Tech Corp., Tokyo, Japan). Pore size distributions in the microstructures were determined by the line counting method using Nano Measure software (version 1.2) [36]. To observe precipitated crystals in the pore walls, porous glass–ceramics were immersed in 10 vol.% HF solution (hydrofluoric acid, FUJIFILM Wako Pure Chemical Corp., Osaka, Japan) for 60 s to remove the glass phase on the surface, and then cleaned with deionized water. Young’s modulus and the hardness of pore walls for the porous LD glass–ceramics were measured using nanoindentation (TI 950 Triboindenter, Omicron Nanotechnology, Tokyo, Japan). The nanoindentation test was operated in load control mode to a prescribed maximum load of 5000 μN, and the loading, dwell, and unloading times were all 5 s. The measured Young’s modulus and hardness values were averaged from 10 nanoindentations per sample. Flexural strengths were investigated by the piston-on-three-ball test [24,37] using a universal testing machine (AGX-10kNVD, Shimadzu Co., Ltd., Kyoto, Japan) equipped with a 5 kN load cell. Specimens were supported by three spherical balls (4.5 mm diameter) positioned 120° apart on a circle (11 mm diameter) and centrally loaded via a flat-surface loading piston 1.4 mm in diameter. The test was performed at a constant crosshead displacement rate of 1 μm/s, and six samples were used for each testing result.

## 3. Results and Discussion

### 3.1. Silicate Structures of Cold-Sintered Pellets

**Figure 2:**
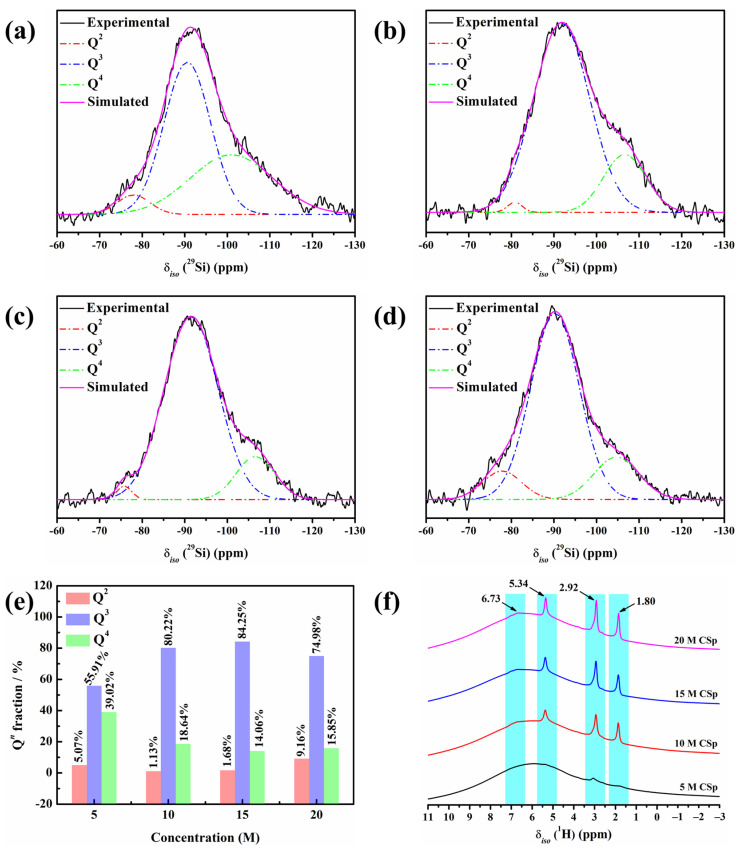
(**a**–**d**) Deconvoluted ^29^Si MAS NMR spectra of 5–20 M CSp; (**e**) the calculated Q*^n^* fractions; and (**f**) ^1^H MAS NMR spectra of silicate structures for 5–20 M CSp.

^29^Si MAS NMR spectra of 5–20 M CSp are shown in Figure 2a–d. All the spectra featured broad and asymmetric bands covering the chemical shifts of the Q^2^, Q^3^, and Q^4^ units [38,39]. Here, Q denotes silicon bonded to four oxygen atoms, and 2–4 represent the amount of bridging oxygen around the silicon atoms [40]. It was revealed that 5–20 M CSp were amorphous [41,42], and their silicate structures consisted of Q^2^, Q^3^, and Q^4^ units. The specific values of chemical shifts (δ*_iso_*) and full widths at half maximum (FWHM) of fitting the Q^2^, Q^3^, and Q^4^ units are listed in Table 2.

The calculated fractions of the Q^2^, Q^3^, and Q^4^ units are presented in Figure 2e. The Q^2^ and Q^4^ unit fractions generally presented declining trends, with the concentration of NaOH solution rising from 5 M to 15 M, whereas an increasing trend was observed for the Q^3^ unit. More formed Si–OH and Si–O^−^Na^+^ groups induced by NaOH solution (Equation (1)) may have possibly accounted for the continual decline in the Q^4^ unit fraction. The result was more condensation reactions (Equation (2)) between hydrated Si–OH groups, which may explain the increase in Q^3^ units and decline in Q^2^ units. In the meanwhile, reverse varying trends were observed for the Q^2^, Q^3^, and Q^4^ units, with the concentration of NaOH solution further rising to 20 M. The slight increment in the Q^4^ unit fraction and mild decrement in the Q^3^ unit fraction may have originated from many more condensation reactions between hydrated Si–OH groups. Moreover, more residual Si–OH and Si–O^−^Na^+^ groups may have possibly responsible for the marked increment in the Q^2^ unit fraction. Based on the Q^2^, Q^3^, and Q^4^ unit fractions, the silicate network connectivity (NC) of 5–20 M CSp was further calculated by Equation (3) [43], as shown in Table 2.

(3) $$\mathrm{Network}\;\mathrm{connectivity}\;(\mathrm{NC})=(4\times \%Q^{4}+3\times \%Q^{3}+2\times \%Q^{2})/100$$

It was found that with the increasing concentration of NaOH solution (5–20 M), the NC values continually decreased from 3.34 for the 5 M CSp to 3.07 for the 20 M CSp, revealing enhanced depolymerization of the silicate structures for the 5–20 M CSp. At the same time, the ^1^H MAS NMR technique was also applied to characterize the silicate structures of the 5–20 M CSp, as shown in Figure 2f. On the one hand, the relative intensities of signals located at 1.80, 2.92, and 5.34 ppm became higher for the 5–20 M CSp, reflecting the existence of more Si–OH groups [44,45]. On the other hand, the relative intensities of signals located at 6.73 ppm also slightly increased with the NaOH concentration rising from 5 M to 20 M, suggesting that more Si–OH…O^−^Na^+^ groups [46,47] appeared for the 5–20 M CSp. It was further confirmed that, when the concentration of NaOH solutions varied from 5 M to 20 M, the silicate structures of the cold-sintered LD glasses featured enhanced depolymerization, which resulted from increasing residual Si–OH and Si–O^−^Na^+^ groups.

### 3.2. Thermal Behaviors of Cold-Sintered Pellets

**Figure 3:**
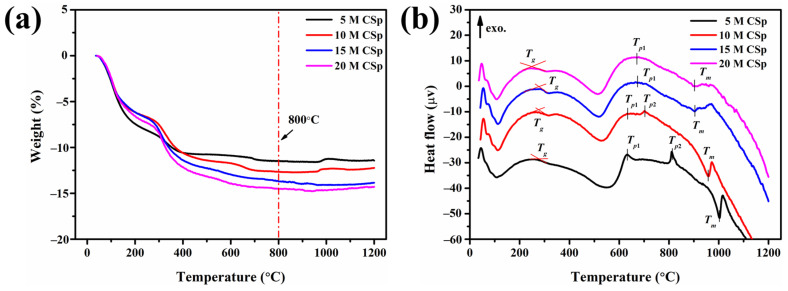
(**a**) TGA and (**b**) DTA curves of 5–20 M CSp.

Thermal behaviors were subsequently detected for the 5–20 M CSp, as shown in Figure 3. The weight losses in the 5–20 M CSp increased from 11.4% to 14.6%, along with the NaOH concentration becoming higher (Figure 3a). As mentioned above, enhanced condensation reactions between residual Si–OH groups and the resulting increased H_2_O vapor were responsible for increasing weight losses [48,49]. Nevertheless, their thermal stabilization temperatures were almost the same, determined to be approximately 800 °C. Meanwhile, various characteristic peaks were also found in DTA curves of 5–20 M CSp, as shown in Figure 3b. The characteristic temperatures are listed in Table 3.

With the increasing concentration of NaOH solution, the glass transition temperature (*T_g_*) decreased from 281 °C to 248 °C, and the melting point (*T_m_*) declined from 1004 °C to 901 °C for 5–20 M CSp. Because *T_g_* and *T_m_* were closely dependent on the polymerization extent and the amount of non-bridging oxygens [31,41] of silicate structures, decreasing *T_g_* and *T_m_* also revealed the enhanced depolymerization of silicate structures for 5–20 M CSp. In addition, with the concentration of NaOH solutions increasing, the exothermic peaks gradually moved to lower temperatures for 5–20 M CSp. For 5 M CSp, two exothermic peaks (*T_p_*_1_ and *T_p_*_2_), located at 630 °C and 812 °C, were found, and *T_p_*_2_ shifted to a lower temperature (703 °C) for 10 M CSp. Moreover, exothermic peaks of both 15 M CSp and 20 M CSp were observed at much lower temperatures, located at 672 °C and 669 °C, respectively. These pronounced exothermic peaks were ascribed to crystallization peak temperatures of cold-sintered LD glasses [41]. Similarly to *T_g_* and *T_m_*, the continually declining crystallization peak temperatures still originated from the enhanced depolymerization of silicate structures for the 5–20 M CSp, due to the fact that depolymerized silicate structures were helpful in decreasing the melt viscosity of the glass matrix and improved the mobility of glass-forming ions during the crystallization process [41]. Based on the thermal analysis results of the 5–20 M CSp, post-annealing temperatures were then determined to be 800 °C. We aimed to completely realize the foaming and crystallizing processes.

### 3.3. Relative Density, Total Porosity, and Phase Structure

**Figure 4:**
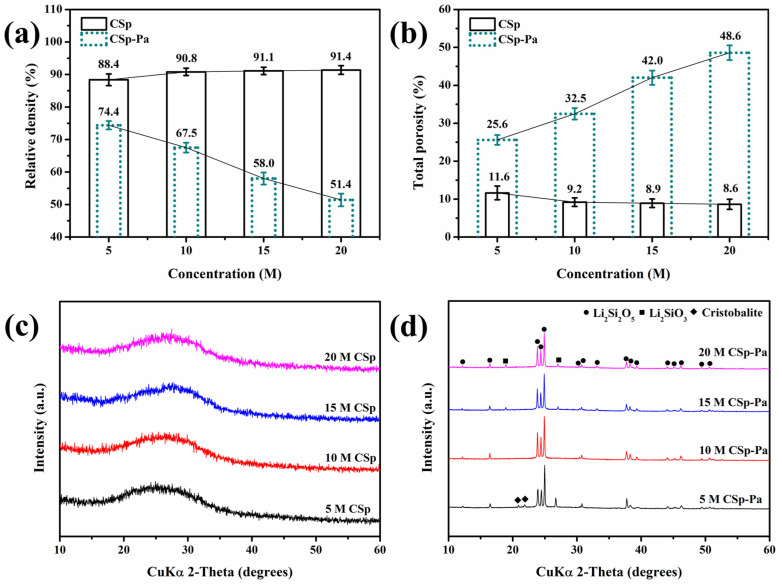
(**a**) Relative densities, (**b**) total porosities, and (**c**,**d**) XRD patterns of 5–20 M CSp/CSp-Pa.

Before and after post-annealing treatment, the relative densities, total porosities, and phase structures of the 5–20 M CSp/CSp-Pa were characterized. These are exhibited in Figure 4. The samples of the 5–20 M CSp possessed very close relative densities and total porosities, whereas the 5–20 M CSp-Pa exhibited declining relative densities and rising total porosities, as shown in Figure 4a,b. Therefore, higher concentrations of NaOH solutions (5–20 M) were used, and lower relative densities and higher total porosities for post-annealed pellets (5–20 M CSp-Pa) were found compared to cold-sintered pellets (5–20 M CSp). On the other hand, amorphous results were found for the 5–20 M CSp, whereas crystalline phases of LD, LM, and cristobalite were found in the 5–20 M CSp-Pa results, as shown in the XRD pattern of Figure 4c,d. The crystallinities and crystalline phase compositions of 5–20 M CSp-Pa are listed in Table 4.

Both the crystallinities of the 5–20 M CSp-Pa and the volume fractions of the LD phase rose, while the volume fractions of the cristobalite and glassy phases declined. Meanwhile, no obvious changes were found in the volume fractions of the LM phase. During the post-annealing process of 5–20 M CSp, the precipitated metastable LM phase was converted into the LD phase through interaction with the cristobalite or glassy phases (Equations (4) and (5)) [42,50]. Although the enhanced depolymerization of silicate structures promoted the precipitation of the LM phase during the crystallization processes [42], the resultant interactions (Equations (4) and (5)) made no obvious changes to the LM phase, along with the markedly increasing fraction of the LD phase and decreasing fractions of the cristobalite and glassy phases.

(4) $${\mathrm{Li}}_{2}{\mathrm{SiO}}_{3}\left(\mathrm{Crystal}\right)+\mathrm{Cristobalite}\left(\mathrm{Crystal}\right)\to {\mathrm{Li}}_{2}{\mathrm{Si}}_{2}O_{5}(\mathrm{Crystal})$$

(5) $${\mathrm{Li}}_{2}{\mathrm{SiO}}_{3}\left(\mathrm{Crystal}\right)+\mathrm{Glassy}\;\mathrm{phase}\to {\mathrm{Li}}_{2}{\mathrm{Si}}_{2}O_{5}\left(\mathrm{Crystal}\right)$$

### 3.4. Microstructrue Evolution

**Figure 5:**
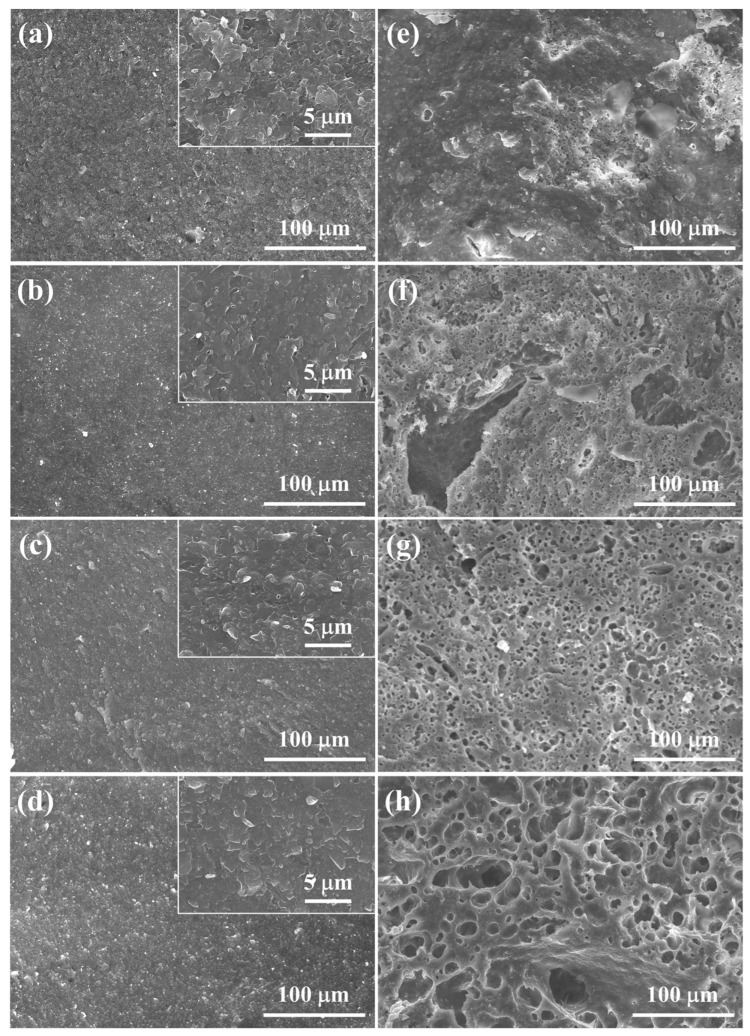
Fracture surfaces of (**a**–**d**) 5–20 M CSp and (**e**–**h**) 5–20 M CSp-Pa.

**Figure 6:**
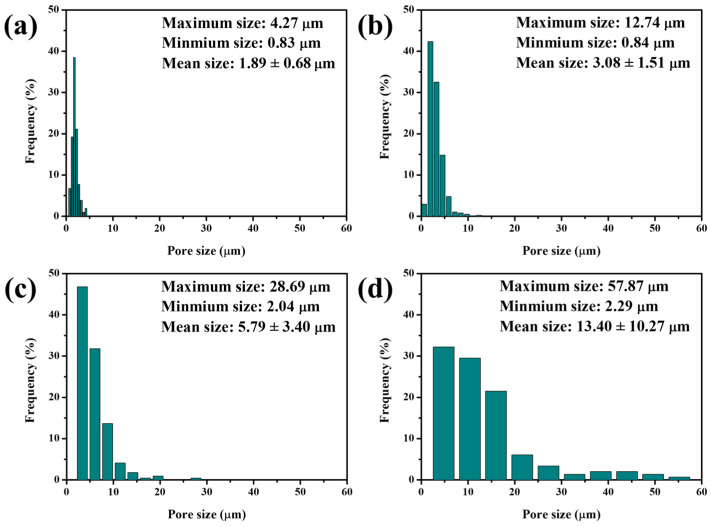
Size distributions of spherical pores formed for porous LD glass–ceramics: (**a**) 5 M CSp-Pa; (**b**) 10 M CSp-Pa; (**c**) 15 M CSp-Pa; (**d**) 20 M CSp-Pa.

Significant differences in relative densities, total porosities, and phase structures between 5–20 M CSp and 5–20 M CSp-Pa reflected that the foaming and crystallizing processes proceeded well with the post-annealing treatment. The fracture surfaces of 5–20 M CSp and CSp-Pa were then characterized to reveal the corresponding microstructure evolutions suggested in Figure 5. The samples of the 5–20 M CSp presented progressively denser microstructures, with the concentration of NaOH solutions increasing. Sintering necks between LD glass particles were clearly found for 5 M CSp (Figure 5a). Meanwhile, pores existing in the fracture surfaces were gradually eliminated during the 10–20 M CSp (Figure 5b–d). Denser microstructures were induced by enhanced dissolution-precipitated processes of raw glass powders under the condition of NaOH solutions with higher concentrations [51]. However, a few spherical pores formed during the 5 M CSp-Pa (Figure 5e), their sizes varying from 0.83 to 4.27 μm, with average values of 1.89 ± 0.68 μm (Figure 6a). The number and size of the formed spherical pores remarkably increased for the 10–20 M CSp-Pa (Figure 5f–h and Figure 6b–d). Spherical pores formed in 20 M CSp-Pa presented the largest average pore size of 13.40 ± 10.27 μm and the widest pore size range of 2.29–57.87 μm. The microstructure changes reflected the formation of more increasingly large spherical pores, accounting for the decreasing relative densities and increasing total porosities for the 5–20 M CSp-Pa in comparison with the 5–20 M CSp.

As mentioned previously, the formation of spherical pores in 5–20 M CSp-Pa was estimated to be caused by H_2_O vapor [27,48,49] originating from condensation reactions (Equation (2)) between residual Si–OH groups in 5–20 M CSp. To be specific, the foaming process of 5–20 M CSp-Pa can be expressed as Equation (6) [32]:

(6) $$-\frac{d\varepsilon }{\varepsilon dt}=\frac{3}{4\eta }(P_{c}-P_{g})$$

where *ε* is the porosity, *η* and *P*_c_ are the viscosity and surface tension of melted glass matrix, and *P*_g_ is the H_2_O vapor pressure inside the formed pores. It is reflected in Equation (6) that not only a higher *P*_g_ than *P*_c_, but also a lower-viscosity *η*, were conducive to pore growth during the foaming process. During the post-annealing process, the increasing condensation reactions between residual Si–OH groups in the 5–20 M CSp and the increased H_2_O vapor resulted in higher *P*_g_ than *P*_c_, and a larger driving force was provided for pore growth during the foaming process. Simultaneously, the restriction of pore growth may have continuously decreased during the foaming process due to the fact that the enhanced depolymerization of silicate structures significantly reduced the melt viscosities in the 5–20 CSp [29,32]. As a result, it is estimated that both increasing condensation reactions between residual Si–OH groups and enhanced depolymerization of silicate structures contributed to the formation of more and increasingly large spherical pores for 5–20 M CSp-Pa in comparison with 5–20 M CSp.

**Figure 7:**
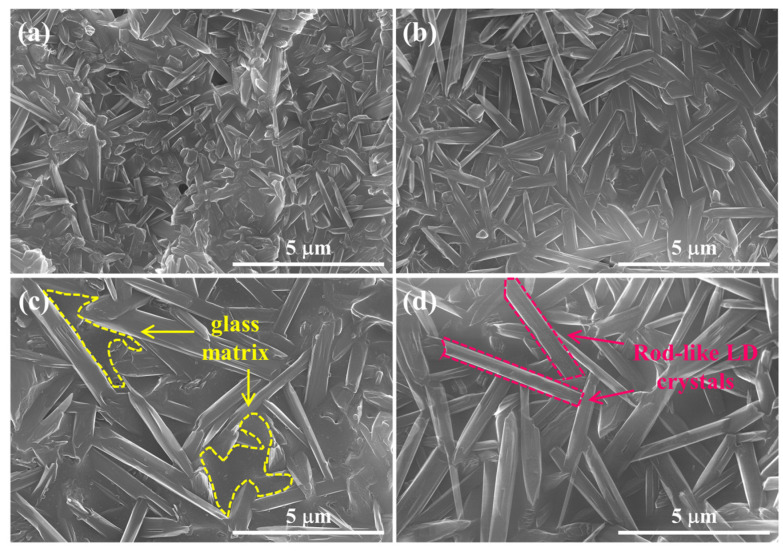
Morphologies of precipitated LD crystals in pore walls of porous LD glass–ceramics: (**a**) 5 M CSp-Pa; (**b**) 10 M CSp-Pa; (**c**) 15 M CSp-Pa; (**d**) 20 M CSp-Pa.

To investigate the morphologies of the crystallized phase, LD crystals precipitated in the pore walls of 5–20 M CSp-Pa were also observed, as presented in Figure 7. LD crystals in the pore walls exhibited closely packed and interlocked morphologies protruding from glass matrices. The LD crystals for the 5 M CSp-Pa had smaller sizes, whereas the 10–20 M CSp-Pa featured larger LD crystals. The detailed statistical results regarding the LD crystal sizes are listed in Table 5. With the concentration of NaOH solution increasing, the average length ( $\bar{L}$ ) and width ( $\bar{W}$ ) of the precipitated LD crystals slightly increased, and the average aspect ratio ( $\bar{R}$ ) also presented a rising trend. The increasing sizes of LD crystals precipitated in the pore walls in the 5–20 M CSp-Pa could be linked with the progressively depolymerized silicate structures in the 5–20 M CSp [29,32].

### 3.5. Mechanical Properties

**Figure 8:**
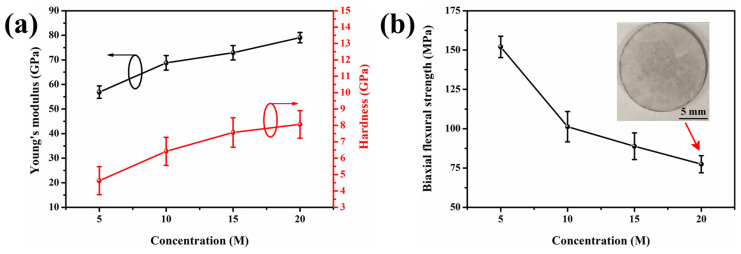
(**a**) The nanoindentation Young’s modulus and hardness of pore walls for 5–20 M CSp-Pa. (**b**) The biaxial flexural strength of 5–20 M CSp-Pa.

The mechanical properties of 5–20 M CSp-Pa were subsequently investigated, as shown in Figure 8. Young’s modulus and the hardness of the pore walls for the 5–20 M CSp-Pa were examined by nanoindentation. Both Young’s modulus and hardness monotonously rose with the varying concentration of NaOH solution (Figure 8a), which was ascribed to increasing volume fractions and aspect ratios of precipitated LD crystals [3,52,53,54,55].

To be specific, with the increasing NaOH concentration, Young’s modulus and hardness varied from 56.9 ± 2.5 GPa to 79.1 ± 2.1 GPa and from 4.6 ± 0.9 GPa to 8.1 ± 0.8 GPa, respectively. It is shown in Figure 8b that the biaxial flexural strength of porous LD glass–ceramics exhibited a sharp decline from 152.0 ± 6.8 to 101.3 ± 9.7 MPa when the concentration of the NaOH solution rose from 5 M to 10 M. This reduction in biaxial flexural strength was attributed to the increasing number and size of spherical pores [36,56]. However, the biaxial flexural strength mildly decreased to 77.4 ± 5.4 MPa when the concentration of the NaOH solution further increased to 20 M. This phenomenon could be ascribed to the presence of more robust pore walls for the 15–20 M CSp-Pa in comparison with the 5–10 M CSp-Pa.

From these results, it can clearly be suggested that this novel technique is beneficial for the preparation of porous LD glass–ceramics with a uniform pore structure as well as a major phase of the LD crystal, which played crucial roles in its practical application. Furthermore, the resultant porous LD glass–ceramics also exhibited proper mechanical properties for its practical application.

## 4. Conclusions

In this study, we successfully prepared porous LD glass–ceramics using CSP associated with the post-annealing technique and investigated the effects of NaOH solution concentrations (ranging from 5 to 20 M) on the foaming and crystallization processes. As the concentration of NaOH solution increased, the silicate structures of cold-sintered LD glasses underwent enhanced depolymerization, resulting in an increase in Si–OH groups. During the post-annealing process, the Si–OH groups further condensed, and then H_2_O vapor formed, which acted as a foaming agent to trigger the formation of spherical pores in the resultant LD glass–ceramics. Compared with denser and amorphous cold-sintered LD glasses, resultant porous LD glass–ceramics exhibited rising total porosities and larger average sizes for the formed spherical pores, which ranged from 25.6 ± 1.3% to 48.6 ± 1.9% and from 1.89 ± 0.68 μm to 13.40 ± 10.27 μm with the increasing concentration of NaOH solutions. At the same time, the volume fractions of LD crystals precipitated in the pore walls increased from 55.75% to 76.85%, and their average aspect ratios varied from 4.18 to 6.53 along with the decreasing LM phase and gradually disappearing SiO_2_ phase. The Young’s modulus and hardness of the pore walls for porous LD glass–ceramics increased from 56.9 ± 2.5 GPa to 79.1 ± 2.1 GPa and from 4.6 ± 0.9 GPa to 8.1 ± 0.8 GPa. The biaxial flexural strength of porous LD glass–ceramics significantly decreased from 152.0 ± 6.8 MPa to 77.4 ± 5.4 MPa. We attribute this decrease in flexural strength to the presence of pores and structural changes induced by the foaming and crystallization processes. Overall, the resultant porous LD glass–ceramics show great potential in the fields of dental restoration, lithium ion batteries, and wastewater treatment. Although further explorations and investigations are necessary in order to fully exploit their capabilities, this work still introduces an easily-operated and eco-friendly technique for preparing porous glass–ceramic materials, offering a promising avenue for future research and applications in advanced materials.

## Figures and Tables

**Table 1:** Notation of the as-prepared pellets.

Samples	Sample Notation
CSP pellets prepared with 5–20 M NaOH solutions	5–20 M CSp
CSP pellets prepared with 5–20 M NaOH solutions after post-annealing treatment	5–20 M CSp-Pa

**Table 2:** Notation of the as-prepared pellets. Chemical shifts (δ*_iso_*), full width at half maximum (FWHM) of fitting Q*^n^* units in ^29^Si MAS NMR spectra for 5–20 M CSp (NC: network connectivity).

Samples	Q^2^		Q^3^		Q^4^		
	δ*_iso_* (ppm)	FWHM (ppm)	δ*_iso_* (ppm)	FWHM (ppm)	δ*_iso_* (ppm)	FWHM (ppm)	NC
5 M CSp	−77.95	7.77	−90.59	10.92	−100.97	19.45	3.34
10 M CSp	−80.82	3.48	−91.79	13.16	−106.63	10.01	3.18
15 M CSp	−75.81	3.47	−91.48	12.73	−106.60	9.07	3.12
20 M CSp	−77.85	9.11	−90.26	11.39	−104.68	10.59	3.07

**Table 3:** Characteristic temperatures of 5–20 M CSp analyzed by DTA.

Samples	Glass Transition Temperature *T_g_* (°C)	First Exothermic Peak *T_p_*_1_ (°C)	Second Exothermic Peak *T_p_*_2_ (°C)	Melting Point *T_m_* (°C)
5 M CSp	281	630	812	1004
10 M CSp	277	633	703	957
15 M CSp	276	672	—	903
20 M CSp	248	669	—	901

**Table 4:** Crystallinities and crystalline phase compositions of 5–20 M CSp-Pa.

Samples	Crystallinity (%)	Crystalline Phase Compositions (%)			
		Li_2_Si_2_O_5_ (LD)	Li_2_SiO_3_ (LM)	Cristobalite	Glassy Phase
5 M CSp-Pa	71.82	55.75	3.96	12.11	28.18
10 M CSp-Pa	72.19	68.56	3.63	—	27.81
15 M CSp-Pa	75.85	71.89	3.96	—	24.15
20 M CSp-Pa	80.74	76.85	3.89	—	19.26

**Table 5:** Average length ( $\bar{L}$ ), width ( $\bar{W}$ ), and aspect ratio ( $\bar{R}$ ) of LD crystals precipitated in pore walls of 5–20 M CSp-Pa.

Samples	5 M CSp-Pa	10 M CSp-Pa	15 M CSp-Pa	20 M CSp-Pa
$\bar{L}$ (μm)	1.04	1.86	3.28	3.24
$\bar{W}$ (μm)	0.25	0.34	0.53	0.50
$\bar{R}$	4.18	5.42	6.19	6.53

## Data Availability

The datasets analyzed or generated during the study are available from the corresponding author upon reasonable request.
